# Advancing Community Mental Health Awareness Through Resident-Led Leadership: A Three-Year Psychiatry Initiative at Hamad Medical Corporation

**DOI:** 10.1192/bjo.2026.11350

**Published:** 2026-06-30

**Authors:** Ahmad Alater, Sazgar Hamad, Shaima Ahmed, Yousaf Iqbal, Majid Alabdulla

**Affiliations:** Hamad Medical Corporation, Doha, Qatar

## Abstract

**Aims:**

The global mental health burden continues to increase, alongside rising community demand for accessible mental health education. While resident-led outreach initiatives are encouraged within graduate medical education, there is limited longitudinal evaluation of their scalability, sustainability, and community impact, particularly within psychiatry and the Middle East. This study aimed to evaluate the design, expansion, reach, and perceived impact of a resident-led Mental Health Awareness programme embedded within leadership training at Hamad Medical Corporation (HMC), Qatar. We hypothesised that a structured resident-led model would demonstrate scalable growth, sustained community engagement, and meaningful perceived benefit.

**Methods:**

A three-year descriptive programme evaluation was conducted for the Leadership Sub-Committee of the Psychiatry Residency Training Program (Mental Health Awareness). The initiative delivered interactive educational sessions, stigma-reduction workshops, and skills-based mental health literacy training tailored to secondary school students and teachers, university students, youth summer programmes, and government-sector staff. Prospective process indicators included number of sessions delivered, annual delivery hours, and diversity of audience sectors. Qualitative narrative feedback from host organisations and participants was systematically collated following each activity. Iterative quality-improvement cycles were implemented to refine content, delivery approaches, and community partnerships.

**Results:**

Programme delivery expanded approximately six-fold over the evaluation period, with annual activity increasing from approximately 25 hours to 150 hours. Engagement evolved from opportunistic school-based sessions to a structured annual outreach calendarencompassing educational institutions, youth programmes, and governmental organisations. Inbound requests and repeat invitations increased consistently year-on-year, demonstrating sustained demand and stakeholder satisfaction. Qualitative feedback highlighted improved mental health literacy, increased willingness to seek professional help, stigma reduction, and practical benefit for educators and organisational staff. Host institutions emphasised cultural relevance, continuity of engagement, and the credibility of resident leadership as key strengths.

**Conclusion:**

Embedding community mental health outreach within structured psychiatry residency leadership training can produce scalable, sustainable, and high-demand public mental health engagement. Progressive expansion in activity volume, diversification of target audiences, and consistently positive stakeholder feedback support institutionalisation and strategic resourcing of resident-led mental health awareness initiatives as a core public mental health function.

